# The Impact of 1030 nm fs-Pulsed Laser on Enhanced Rayleigh Scattering in Optical Fibers

**DOI:** 10.3390/s24185980

**Published:** 2024-09-15

**Authors:** Bogusław Szczupak, Mateusz Mądry, Marta Bernaś, Paweł Kozioł, Krzysztof Skorupski, Gabriela Statkiewicz-Barabach

**Affiliations:** 1 Department of Telecommunications and Teleinformatics, Faculty of Information and Communication Technology, Wroclaw University of Science and Technology, Wybrzeże Wyspiańskiego 27, 50-370 Wrocław, Poland; 2 Department of Optics and Photonics, Faculty of Fundamental Problems of Technology, Wroclaw University of Science and Technology, Wybrzeże Wyspiańskiego 27, 50-370 Wrocław, Poland; marta.bernas@pwr.edu.pl; 3 Department of Field Theory, Electronic Circuits and Optoelectronics, Faculty of Electronics, Photonics and Microsystems, Wroclaw University of Science and Technology, Wybrzeże Wyspiańskiego 27, 50-370 Wrocław, Poland; pawel.koziol@pwr.edu.pl; 4 Department of Computer and Electrical Engineering, Lublin University of Technology, Nadbystrzycka 38 D, 20-618 Lublin, Poland; k.skorupski@pollub.pl

**Keywords:** Rayleigh scattering, optical fibers, optical frequency domain reflectometer, fiber Bragg gratings, distributed optical fiber sensors, light reflection, optical fiber sensors

## Abstract

This article presents a comprehensive study on the impact of irradiation optical fiber cores with a femtosecond-pulsed laser, operating at a wavelength of 1030 nm, on the signal amplitude in Rayleigh scattering-based optical frequency domain reflectometry (OFDR). The experimental study involves two fibers with significantly different levels of germanium doping: the standard single-mode fiber (SMF-28) and the ultra-high numerical aperture fiber (UHNA7). The research findings reveal distinct characteristics of reflected and scattered light amplitudes as a function of pulse energy. Although different amplitude changes are observed for the examined fibers, both can yield an enhancement of amplitude. The paper further investigates the effect of fiber Bragg grating inscription on the overall amplitude of reflected light. The insights gained from this study could be beneficial for controlling the enhancement of light scattering amplitude in fibers with low or high levels of germanium doping.

## 1. Introduction

Optical frequency domain reflectometry (OFDR) has been extensively explored in different sensing applications and characterization of optical fiber components [1,2,3,4]. The principle of operation in OFDR is based on the analysis of Rayleigh-backscattered light to obtain high-resolution spatial information about the optical fiber. Rayleigh scattering occurs in each fiber due to the interaction of photons with small-scale inhomogeneities or fluctuations in the refractive index of the medium. The amplitude of Rayleigh backscatter is higher than other scattering mechanisms; however, its level in standard single-mode fiber (SMF) is still weak, which could affect the accuracy of OFDR measurements. When the signal-to-noise ratio (SNR) is increased, the detection sensitivity can be improved, expanding the potential applications and capabilities of the OFDR technique. Furthermore, modifying the reflection amplitude of fibers can be used to implement the scattering level multiplexing (SLMux) scheme [5,6], allowing the distinction of different lines based on varying levels of backscattered light, for instance, for sensing purposes.

Numerous methods to increase the amplitude of reflected light have been proposed in the literature [7,8,9,10,11,12,13,14,15]. A particular technique involves post-fabrication treatment, which includes the irradiation of fibers with light [7,8,9]. For example, Wang et al. have reported a notable increase in scattered light by approximately 16.5 dB in terrestrial telecommunication optical fibers when employing a laser with a repetition rate of 250 kHz at 800 nm [7]. UV irradiation (213 nm laser) has also been used to enhance amplitude backscattering to approximately 20 dB [8]. In another study, hydrogen-loaded fibers were subjected to ultraviolet exposure using a 266 nm laser for the creation of fiber Bragg grating (FBG) [9]. Such a modification resulted in an increase in Rayleigh scattering by 38 dB, which can be related to changes in the material properties. It is important to note that the scanned wavelength range differed from the Bragg wavelength to eliminate its reflection. Another study concerns an investigation of UV irradiation into hydrogen-loaded SMF and an analysis of its effect on strain measurement [10]. The distance between the phase mask and fiber, laser energy, and the moving velocity of fiber during laser exposure have been thoroughly analyzed. The maximal achieved enhancement of Rayleigh scattering was 37.3 dB. Furthermore, an alternative approach to laser treatment involves the use of optical fibers with various structures [11,12,13,14,15,16]. Double-clad fibers have been investigated and shown to exhibit an increased level of backscattered light [11]. Our research examining different fibers doped with germanium has revealed that modifications in germanium concentration within the fiber cores can significantly improve the amplitude of reflected light [12]. In addition, the incorporation of nanoparticles in fibers has been demonstrated to enhance the amplitude of light reflection [13,14,15,16]. Magnesium oxide (MgO) nanoparticles, both around and within the core [13], as well as calcium-based nanoparticles, have been shown to boost Rayleigh scattering [14]. Another approach used gold nanoparticles to effectively increase the reflection [15]. The optical fibers doped with zirconia nanoparticles have also achieved improvements in light scattering levels of approximately 40 dB [16]. The application of femtosecond laser technology has shown significant potential in enhancing the performance of optical fibers for distributed fiber sensors. One study employed a femtosecond laser to enhance the Rayleigh backscattering profile of a standard telecom fiber, achieving an improvement in the SNR for OFDR by over 40 dB. This enhancement enabled effective distributed strain measurements using a low-cost tunable laser, achieving a spatial resolution of 4.8 cm and a root mean square accuracy of less than 2.70 με [17]. Another study demonstrated the use of femtosecond laser inscription to fabricate an array of weak FBGs in SMF. This approach allowed for strain measurements up to 10,000 με with a spatial resolution of 5 mm, improving the cross-correlation coefficient to 0.9 and enabling precise position deviation measurements under significant strain [18]. However, to date, there have been no literature reports addressing the influence of near-infrared femtosecond-pulsed laser irradiation on scattered and reflected light in OFDR setups concerning varying levels of germanium concentration in fiber cores. This paper, for the first time to the best of our knowledge, presents a comprehensive study of 1030 nm fs-pulsed irradiation on the amplitude of backscattered and reflected light carried out in low- and high-doped germanium optical fibers. We aimed to investigate whether there is a relationship between pulse energy and enhanced amplitude for fibers with different germanium concentrations (low and high), and if so, what type of relationship it is. The influence of heavily Ge-doped optical fiber (UHNA7) has been investigated and compared with SMF-28. The dependence of laser pulse energy on backscattering and reflected amplitude based on different fibers has been exactly examined. The characteristics between enhanced light scatter level and laser pulse energy for low- and high-Ge-doped fibers are different. The increase in scatter level amplitude change is higher for UHNA7; however, it is still possible to prominently enhance the scattering level magnitude for low-Ge-doped fiber (SMF-28). The observable phenomenon for illumination by a near-IR laser is different from exposure to UV and provides an opportunity to create an enhanced scattering level in different fibers without the need for a hydrogen-loading process or photosensitive fibers. Moreover, this study opens up a relatively easy way to enhance scattering in fibers, which could be used in the SLMux scheme.

## 2. Materials and Methods

**Figure 1:**
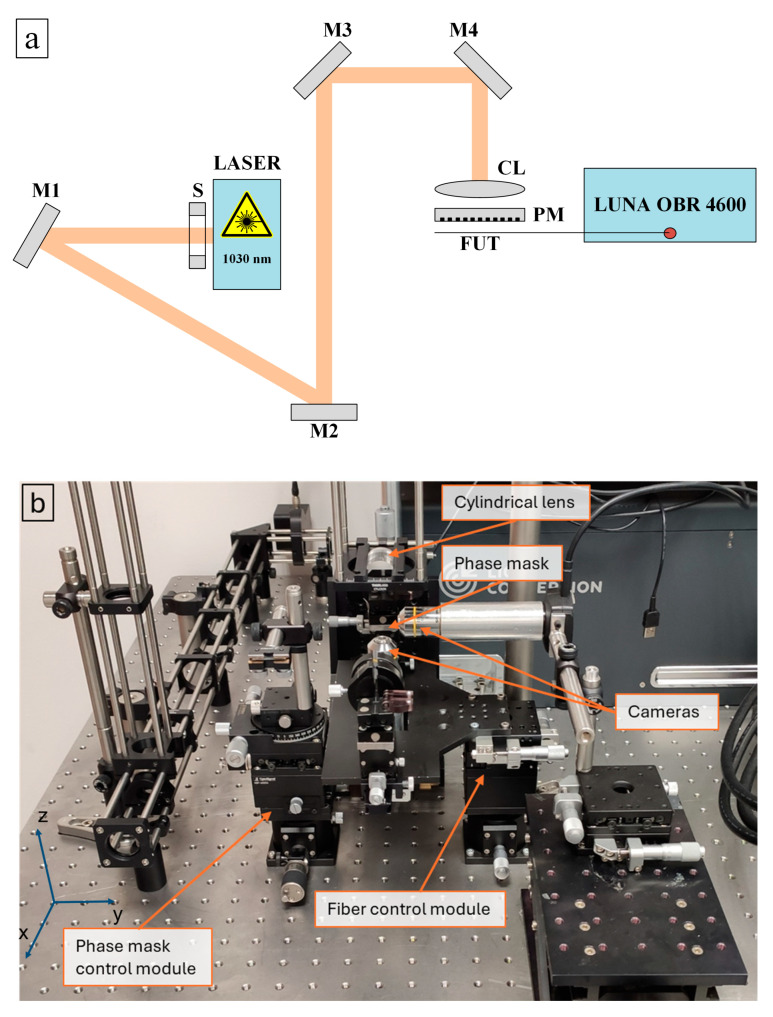
(**a**) The scheme and (**b**) photo of the setup for 1030 nm laser irradiation into optical fibers. LASER: 1030 nm fs-pulsed laser, S: shutter, M: mirrors, CL: cylindrical lens, PM: phase mask, FUT: fiber under test, LUNA OBR 4600: Rayleigh-based OFDR.

The light irradiation was performed using a femtosecond laser Pharos PH1-SP-1mJ (Light Conversion, Vilnius, Lithuania) operating at 1030 nm. The linearly polarized laser beam was focused on the fiber through the phase mask by a plano-convex cylindrical lens with a focal length of 30 mm. The setup is illustrated in Figure 1 (schematic view and corresponding photo).

In the experiment, we used the uniform phase masks from (Ibsen Photonics, Farum, Denmark) with periods of 2150 nm and 2170 nm customized for 1030 nm writing wavelength. These masks allow for the inscription of the 2nd-order gratings with the primary Bragg peaks closed to λ_B_ = 1550 nm. Its diffraction efficiency for the zero order was around 4% when +1/−1 orders were above 40%. The measured values of the efficiency for all diffraction orders are gathered in Table 1.

To investigate the effect of femtosecond laser irradiation on the Rayleigh scattering amplitude in the examined fibers, measurements were carried out for different pulse energy values. The maximum output power and pulse energy of the laser are 6 W and 1 mJ, respectively. All measurements were performed with the same repetition rate of 6 kHz. The pulse duration of the laser used was 190 fs, and it was kept constant while only the output power was varied. The examined fibers were SMF-28 as a conventional telecom fiber with a low Ge concentration in its core and UHNA7 as a high-NA fiber heavily doped with Ge. The important parameters of the investigated fibers are included in Table 2.

In the investigated fibers, the core diameters differ significantly, as well as MFDs and germanium concentrations. In SMF-28 and UHNA7, the germanium content is 3.5 mol% and about 40 mol%, respectively. The germanium concentrations for the investigated fibers were obtained based on previously measured values of NA [12,19].

**Figure 2:**
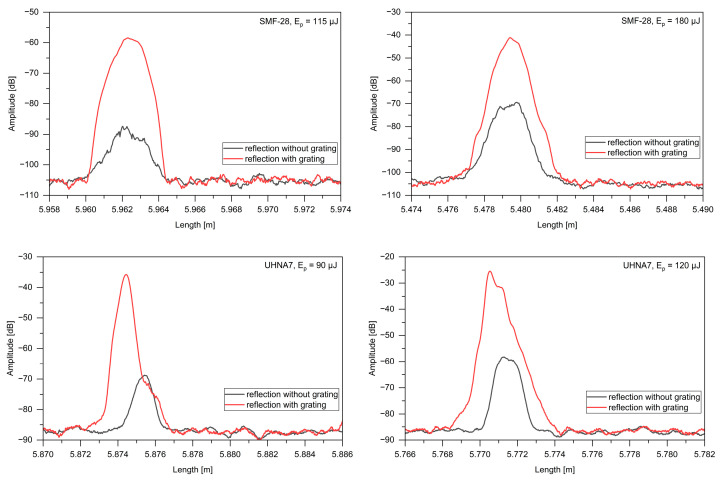
The registered amplitudes of reflected light for pulse energies of 115 μJ and 180 μJ for SMF-28 as well as 90 μJ and 120 μJ for UHNA7 with respect to full (with grating) and narrower (without grating) wavelength ranges.

Before starting the exposure, the camera placed under the fiber was moved in the z-plane so that the image of the beam (of low power), after passing through the cylindrical lens and the phase mask, was focused. The fiber was then moved in the x-plane so that the fiber core was located at the beam location and in the z-plane so that the image of the beam, after passing through the fiber, was focused again. This procedure allows the illumination beam to be focused on the fiber core. During exposure, the fiber was additionally moved step-by-step along the z-axis by approximately ±2 µm, observing the scattering amplitude, to precisely place the core in the beam focus. The position of the lens and the mask relative to the lens remained constant throughout the exposure process, and the distance between the fiber and the phase mask was 200–400 µm. Rayleigh scattering amplitude measurements were performed in the spatial domain using an OFDR Luna OBR 4600 reflectometer (Luna, Roanoke, VA, USA). For each pulse energy of the laser, the fibers were irradiated in three different places so that the maximum amplitude level could be found and to check the repeatability of the irradiation. We tried to ensure that the exposure time for each section was the same, approximately 1 min. The quality of exposure was also verified by observing the reflection spectrum of the Bragg grating in the frequency domain. After illuminating a section of fiber, the amplitude was recorded for two different reflectometer scanning wavelength ranges. This made it possible to register changes in the scattering amplitude in the exposed section when the reflectometer scanning range did not cover the Bragg wavelength of the fabricated grating (1545.45–1566.45 nm). At the maximum scanning range (1545.45–1588.03 nm), the registered amplitude additionally took into account the reflection from the Bragg grating. The examples of recorded amplitudes for both investigated fibers and different scanning wavelength ranges, i.e., full and narrower, are shown in Figure 2. The length of the exposed section with increased amplitude is 3 mm, which corresponds to the diameter of the laser beam. The distribution of the amplitude change corresponds to the Gaussian power distribution in the beam cross-section. The spatial resolution during measurements was set at 0.15 mm.

## 3. Results and Discussion

**Figure 3:**
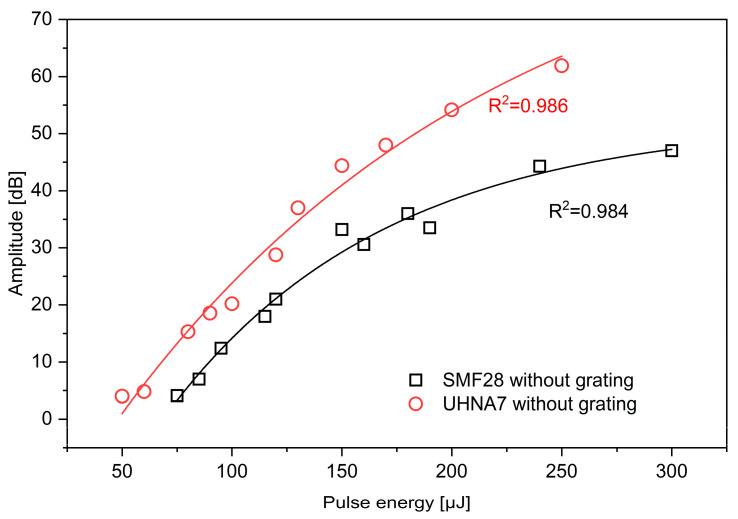
The enhancement of light amplitude as a function of pulse energy for SMF-28 and UHNA7 fibers. The measurements were performed for the narrower wavelength range of the laser in the OBR setup (1545.45–1566.45 nm).

In OFDR setups, the amplitude of scattered light depends on the inhomogeneities in fiber and varies with refractive index changes. For example, significant backscattering occurs at the boundaries of different materials, such as air and glass. In practical applications, the enhancement of signal amplitude is crucial and improves the capabilities of OFDR operation. This could be achieved by inducing variations in the refractive index or by creating micro-damages within the fiber. Thus, in this section, the results of amplitude enhancement for different fibers (low- and high-doped germanium) using 1030 nm irradiation are presented. However, to eliminate the influence of the Bragg resonance on the reflected light, the analysis was limited to a narrower wavelength range. This approach implies measuring the proper increase in the backscattering light amplitude. The change in amplitude enhancement for SMF-28 and UHNA7 as a function of pulse energy is presented in Figure 3. It is clearly shown that in the case of the UHNA7, less laser pulse energy is needed to increase the backscattered amplitude level recorded by the reflectometer. This is probably due to the high level of germanium dopant in the core of UHNA7, which results in a higher absorption coefficient than the low-germanium-doped SMF-28. This is straightforwardly related to the higher light absorption of germanium than of silica at 1030 nm [20]. We believe that both absorption and micro-damages should be accounted for enhancement. It is also clear that the amplitude increases with increasing laser pulse energy. For lower values of pulse energy, the enhancement of scattering level is possible much faster in the case of UHNA7 than SMF-28. Moreover, the amplitude enhancement decreases for SMF-28 at higher values of pulse energy, which is observably different from UHNA7.

The fitting functions for the relationship between enhanced scattering amplitude and laser pulse energy for SMF-28 and UHNA7 are as follows:

(1) $$y_{SMF-28}=-104.21346\cdot e^{\left(\frac{-x}{99.81744}\right)}+52.42257\;$$

(2) $$y_{UHNA7}=-122.39444\cdot e^{\left(\frac{-x}{175.5282}\right)}+93.04989$$

**Figure 4:**
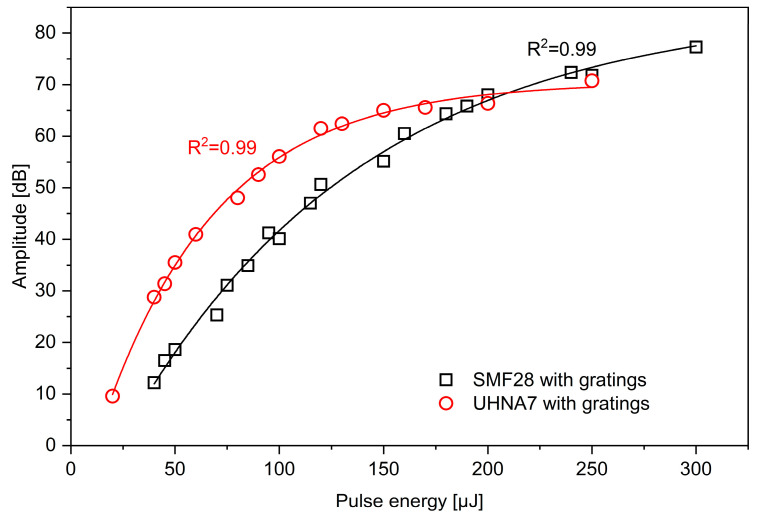
The enhancement of light amplitude as a function of pulse energy for SMF-28 and UHNA7 fibers. The measurements were performed for the full wavelength range of the laser in the OBR setup (1545.45–1588.03 nm).

The R^2^ for SMF-28 and UHNA7 was 0.984 and 0.986, respectively. Based on fitting results, the output values of UHNA7 decay more slowly as a function of increasing laser pulse energy and approach a higher asymptote compared to SMF-28. The maximum enhancement of backscattered amplitude is about 61.9 dB for UHNA7 at 250 μJ and 47 dB at 300 μJ for SMF-28. The reference backscattered amplitude level (without enhancement) for the highly doped UHNA7 fiber was −87.5 dB, while for the SMF-28 fiber, a significantly lower value of −105.5 dB was observed. Thus, the enhanced amplitude was −25.6 dB for UHNA7, which is much higher than in the case of SMF-28 (−58.5 dB). To show total reflection amplitudes and the contribution of the grating, measurements were performed for the full wavelength range of the laser. Figure 4 illustrates the enhanced value of reflected amplitude involving both backscattering and grating resonance as a function of pulse energy for the investigated fibers. According to Figure 4, the amplitude changes nonlinearly in both cases. The fitting of the data shows an exponential relationship for both types of fibers (R^2^ = 0.994 for SMF-28 and R^2^ = 0.997 for UHNA7):

(3) $$y_{SMF-28}=-103.55816\cdot e^{\left(\frac{-x}{115.13235\;}\right)}+85.14251\;$$

(4) $$y_{UHNA7}=-86.38334\cdot e^{\left(\frac{-x}{56.35048}\right)}+70.52971$$

Based on this set of fitting results, the output values of UHNA7 decay more quickly as a function of increasing laser pulse energy and approach a lower asymptote compared to SMF-28. However, at lower values of pulse energy, UHNA7 has a higher enhancement of reflection than SMF-28. For instance, at 50 μJ, the observed enhancement in amplitude was 18.63 dB for SMF-28 and 35.5 dB for UHNA7. At 100 μJ, the enhancement reached 40 dB for SMF-28 and 56.05 dB for UHNA7.

**Figure 5:**
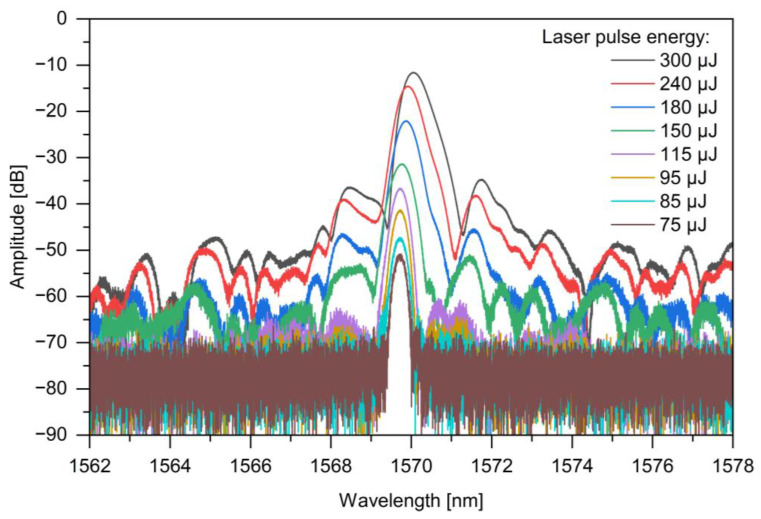
The exemplary inscription of the grating due to different values of pulse energy in SMF-28.

Finally, the maximum observed amplitude enhancement was 70.76 dB for UHNA7 and 77.27 dB for SMF-28. As a consequence, the highest improved amplitude was −16.74 dB for UHNA7 and −28.23 dB for SMF-28. In the case of the UHNA7 fiber, at higher laser pulse energy values, the enhanced amplitude reaches a huge level, which potentially exceeds the optimal operating range of the detector recording the backscattered amplitude, and this may cause faulty operation of the reflectometer. Therefore, the values of enhanced amplitude for the UHNA7 fiber may not be correct and may be underestimated. Furthermore, it is worth emphasizing that when irradiation was conducted with pulse energy greater than 200 μJ, damage to the phase masks was observed at the site of irradiation. The higher values obtained during these measurements and depicted in Figure 4 relate to the contribution of the reflection from the Bragg grating structure. An example of written FBGs in SMF-28, for pulse energy values from 75 μJ to 300 μJ, is shown in Figure 5. The higher the pulse energy of laser used, the greater the reflectivity and widening of the FBG spectrum obtained. The red shift of the Bragg wavelength was observed during the irradiation process, which indicates an increase in the average index change, and this is normal behavior for FBGs of Type I [21]. A shift in the Bragg wavelength towards longer wavelengths was observed in both fibers. In the case of UHNA7, compared to SMF-28, this shift was much larger and probably caused by the higher concentration of germanium in the core and its higher thermo-optic coefficient. Nevertheless, opposite to UV radiation, where hydrogen loading or photosensitive fiber is needed, herein, illumination of near-IR can be adjusted to enhance backscattering light amplitude or create a specific grating even in conventional SMF-28. The use of standard fiber has an economic advantage because of the higher cost of photosensitive fibers.

Moreover, such an adjustment of backscatter amplitude could be used to utilize the SLMux scheme, which was presented by Tosi et al. [22]. This approach allows the creation of a network of sensing units by the usage of a light splitter and dividing the signal into different branches. Unlike the traditional sensing line or time division multiplexing (TDM) scheme, SLMux could be beneficial for the truly simultaneous measurement of different branches. An example of such a setup has been presented for an epidural catheter for the simultaneous measurement of multiple fibers by OFDR and SLMux [23].

## 4. Conclusions

In this paper, a comprehensive study on the influence of fs-pulsed irradiation on the Rayleigh-based backscattering amplitude profile in low- and high-germanium-doped fibers is thoroughly presented. The results reveal that the change in amplitude depends on the type of fiber and its germanium concentration. For heavily germanium-doped fiber, the amplitude significantly increases at lower pulse energy levels, with the change being less pronounced at higher pulse energy levels. For lower germanium-doped fiber, the amplitude changes slowly at lower values of laser pulse energy. However, both the scattering amplitudes of SMF-28 and UHNA7 could be significantly enhanced. This study clearly demonstrates that using a standard SMF-28, without hydrogen loading, the amplitude could be enhanced as well as for heavily germanium-doped fibers. However, the main enhancement could be achieved in UHNA7 if needed. The findings are essential because they demonstrate the possibility of adjusting the amplitude of backscattered light independently of germanium concentration. This process could be used to enhance the amplitude of light scattering in order to implement the SLMux scheme.

## Figures and Tables

**Table 1:** Diffraction efficiencies measured for the phase masks with periods of 2150 nm and 2170 nm used for fiber irradiation.

Mask Period	Diffraction Order m =	0	+1	−1	+2	−2
2150 nm	**Efficiency [%]**	4.5	40.8	41.8	6.4	6.5
2170 nm	**Efficiency [%]**	4.2	42.5	43.4	4.9	4.9

**Table 2:** The parameters of selected fibers.

Type of Fiber	SMF-28	UHNA7
Core/Cladding Diameter [μm]	8.2/125	2.4/125
Mode Field Diameter (MFD) @1550nm [μm]	11.1	3.3
Core NA (catalog card)	0.14	0.41
Core NA (measured)	0.149	0.47
Germanium Dopant Level [mol%]	3.5	39.8

## Data Availability

The original contributions presented in the study are included in the article, and further inquiries can be directed to the corresponding authors.
